# Modification of Erroneous and Correct Digital Texts

**DOI:** 10.3390/e26121015

**Published:** 2024-11-23

**Authors:** Mária Csernoch, Carolin Hannusch, Piroska Biró

**Affiliations:** 1 Faculty of Informatics, University of Debrecen, 4032 Debrecen, Hungary; hannusch.carolin@inf.unideb.hu (C.H.); biro.piroska@inf.unideb.hu (P.B.); 2 Faculty of Economics, Socio-Human Sciences and Engineering, Sapientia Hungarian University of Transylvania, 530104 Miercurea-Ciuc, Romania

**Keywords:** end-users, digital texts, entropy, waste, effectiveness, problem-solving strategies

## Abstract

The end-user paradox and the illusion of digital prosperity reveal the contradictory situation in which both non-professional and professional computer scientists and engineers seem satisfied with digital development but unaware of the magnitude of waste generated by end-users and their digital artifacts. To measure this waste and to reveal end-users’ problem-solving strategies, our research group set up an objective measuring system that can calculate the entropy of digital texts (EDT). To calculate EDT, a testing process of 53 participants was launched where erroneous and correct natural language digital texts were modified according to the requirements of the tasks. It was found that erroneous documents require more time and information to be modified, which implies that waste is generated by handling these documents. It was also found that when the problem-solving processes are broken down into atomic steps, EDT can reveal uncertainty and idleness, which further increases waste. The goals of the present paper are to call attention to (1) the hidden waste generated by billions of end-users and its consequences, (2) educational approaches and general ignorance which have led to these low-level results, and (3) the need to set up a standard evaluation system for further analyses.

## 1. Introduction

End-user computing is the “dark matter” of informatics and computing [1,2]. Professional informatics and computer science (CS) cannot handle it for various reasons including, (1) the huge number of end-users, approaching in number the population of the entire world, including CS professionals in an end-user role (detailed in [3]); (2) the lack of effective and efficient CS-based digital problem-solving approaches for handling end-users and their digital artifacts [4,5]; (3) the position of end-users in the digital world and the contradictory approaches in relation to this [6,7,8,9,10,11,12]; (4) the wide variety of end-users; (5) misleading testing and self-assessment evaluation techniques and systems [13,14,15,16,17,18,19,20,21,22,23,24]; (6) widely accepted and applied tool-centered learning-teaching approaches; and (7) the ignorance and negligence of computer education theories in general [25,26], and the results of objective measuring systems in particular [3,27,28,29].

The misconception connected to end-users, the misplacement of end-users in the CS realm, the lack of a human-centered end-user philosophy, the underdevelopment of digital learning-teaching strategies, and the ignorance and negligence of waste generated by end-users and their digital artifacts has led to the end-user paradox and the illusion of digital prosperity. The end-user paradox states that the more underdeveloped end-users are, the more errors they make, and thus the more resources are required to handle these errors and their digital artifacts. The abundance of digital tools, including both hardware and software, creates the idea—the illusion—that we are in a digitally advanced state and that the possession of tools [30,31,32,33] and their handling are enough to solve digital and digitized problems [34,35]. On the other hand, it has been proven that having tools and collecting them in great abundance does not solve digital problems but is rather a distraction [36,37,38,39,40] that does not serve the development of their owners but is a hindrance.

The objectives connected to end-user computing are as follows, without aiming to cover all aspects of the problem:

- End-users generate erroneous documents [1,41,42,43,44,45,46,47,48,49] and huge waste in their document handling processes [2,27,28,29];
- In spreadsheeting, waste can surface and result in immediate losses [1,46]. However, in general, end-user waste remains unnoticed, unrecognized, ignored, neglected, overlooked, etc. It is silent waste;
- Digital text management processes, including word processing, presentation and webpage management, and handling text in a data management and programming context, generate huge hidden losses [3,27,28,29]. The volume of these losses is barely studied and revealed;
- Digital end-user artifacts (data files) also generate huge losses when their creation and the need for modification surface [2,27,28,29];
- The end-user paradox and the illusion of digital prosperity take its toll [29];
- End-user computing is on the periphery of CS and is barely connected to any other CS-related skills, abilities, competencies, and knowledge, despite the fact that it fulfills all the requirements of CS-related problem-solving [6,31,50,51,52,53].

Considering these objectives, a previously introduced method, namely the entropy of digital text (EDT) [27] is applied to reveal end-users’ behavior in digital environments with various target conditions. The entropy of natural language digital text is based on Shannon’s original entropy theory [54,55] but tuned to the characteristics of processes which handle these artifacts. To calculate EDT, a text-management process is recorded and analyzed based on the recorded video file [3,4]. The activities (steps, actions) of a process are identified and a time stamp (the length of the step) is assigned to each step. Based on this stamp, the probability of the activity is calculated. After collecting all these stamps, the entropy of a process is calculated, which is the EDT of a digital process [27].

The purpose of the present paper is—based on the EDT values—to shed light on how effectively and efficiently end-users can work in natural language digital texts, what problem-solving strategies they apply to handle fundamental modification processes in word processing, and how digital reading—the understanding and interpretation of the graphical interface—supports or impedes workflow [56,57,58,59,60,61].

## 2. Materials and Methods

To set up and validate a measuring system which can guide researchers and teachers to detect end-user problem-solving strategies, a method previously introduced by Nagy and Csernoch and detailed in [3] was adapted. Within this framework, a test is carried out where:

- Participants solve quasi-guided modification tasks in erroneous and correct MS Word documents (four documents altogether);
- Participants’ activities are recorded in text and video forms by a dedicated application called ANLITA (Atomic Natural Language Input Tracker Application) [3];
- Our research group used the modified MS Word documents and the recorded log files as data sources in the planned evaluation system.

In the following sub-sections, the materials, methods, sample, and evaluation process, along with our hypotheses and the ethical issues relevant in the present situation, are discussed in detail. Following this background information, the results of our study are presented.

### 2.1. Materials

**Figure 1:**
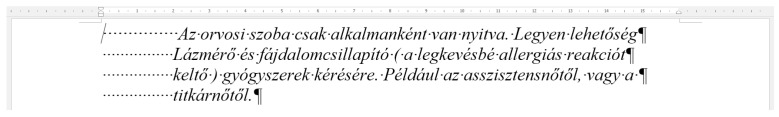
The test string of the original erroneous document in Hungarian.

**Figure 2:**
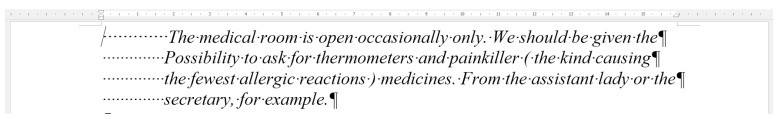
The test string of the original erroneous document in English.

For the test, a short paragraph of an erroneous Word document and its correct correspondent were selected (Figure 1 and Figure 2) [62]. The document is from the private collection of the research group and is authentic, having been created by an unidentifiable middle-school student. The original language of the text is Hungarian (Figure 1). This piece of text was previously analyzed [3,62] and found to consist of both quantitative and qualitative errors [3,29,62,63]. The descriptive statistic of the selected string (paragraph) is presented in Table 1. The data of the table and the figures reveal that the major qualitative errors belong to the layout category [3,62], where the one-paragraph string is broken into four paragraphs and the left indentation is imitated by multiple Space characters (Figure 1 and Figure 2). It is obvious that the selected string does not fulfill the requirements of the definition of the properly edited text [3,29], since the text is not invariable to modification; consequently, any change to the original text requires further activities to reach the original or a similar appearance of the text.

**Figure 3:**
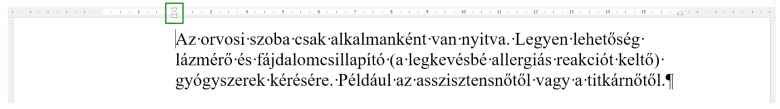
The test paragraph of the correct document in Hungarian. The green rectangular on the ruler indicates the left indentation of the paragraph.

On the other hand, the proper string consists of only one paragraph and the left indentation is correctly formatted (Figure 3), which is indicated by the indentation icons on the ruler. Consequently, any change to the text does not require further correction to reach the desired appearance. It must also be mentioned that the original document has further errors which are corrected but not involved in the test [2] (Figure 1 vs. Figure 3).

### 2.2. Methods

**Figure 4:**
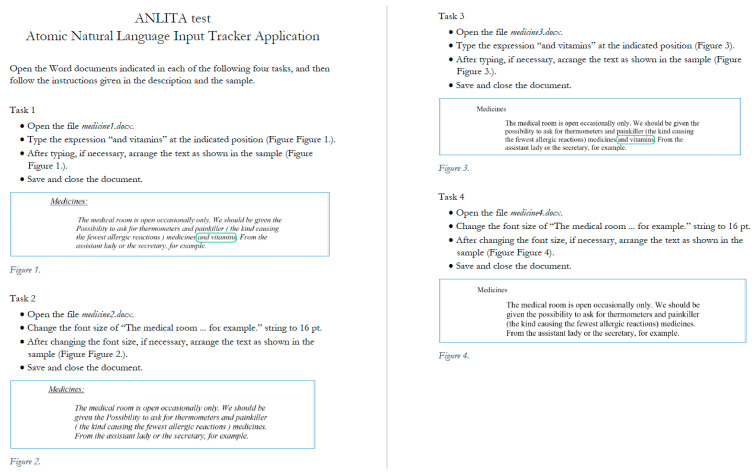
The instruction PDF explaining the four tasks and presenting figures as a guideline. The blue rectangles indicate the pieces of text which should be modified, and the green rounded rectangles indicate the text which should be typed.

To carry out the test, both the erroneous and the correct documents were provided twice (Tasks 1–4) along with an instruction PDF file (Figure 4) [62]. At the beginning of the testing process, the supervisor describes the tasks, the process, and the goals of the test in detail. After accepting the test conditions, the participants open the PDF file (Figure 4) on their computer to make it available during the test. The PDF file for each task explains the task in a four-step algorithm and provides a figure presenting the approximate output.

In Tasks 1 and 2, the erroneous documents are used, while Tasks 3 and 4 involve the correct ones. In Tasks 1 and 3, a 13-character long string and one Space should be typed, while in Tasks 2 and 4, the font size of the text should be changed to 16 pt [62].

When the participant has become familiar with the tasks, they start the recording, which is carried out using the ANLITA (Atomic Natural Language Input Tracker Application) application detailed in Nagy and Csernoch [3]. The output of the recording consists of two files. One of them is a text file, which tracks all the keyboard and mouse events, while the other is a video file, which records the complete process visible on the computer screen. Furthermore, the modified and saved Word files are also collected for further analyses [62]. Altogether, six output files belong to one participant. All these files are considered for evaluation on different levels and with various methods, combining quantitative and qualitative analyses (triangulation) [64,65].

The present paper focuses on the entropy of the processes. We are interested in tracking and revealing the problem-solving strategies and actions the participants applied during the processes and information content of these steps. We also want to find an explanation of how entropy is affected by these solutions and how these pieces of information can serve us in building educational strategies [28]. Furthermore, previous studies revealed that handling and managing erroneous documents generates waste [3,27,28,29,62]. We are interested to see whether such a short text and the atomic steps needed to complete the tasks can reveal differences between erroneous and correct documents.

To find answers to our research questions, it was mainly the recorded videos which were analyzed. Occasionally, the other sources were used when further data were needed or the video was not trackable. One such data point is whether the tasks are completed or not. The video can reveal this characteristic of the documents, but for automated analyses, modified Word documents were used. On the other hand, whether or not the participants displayed the non-printable characters on the screen is only detectable in the video, since it depends on the status of the previously used document in Word [62].

### 2.3. Sample

**Figure 5:**
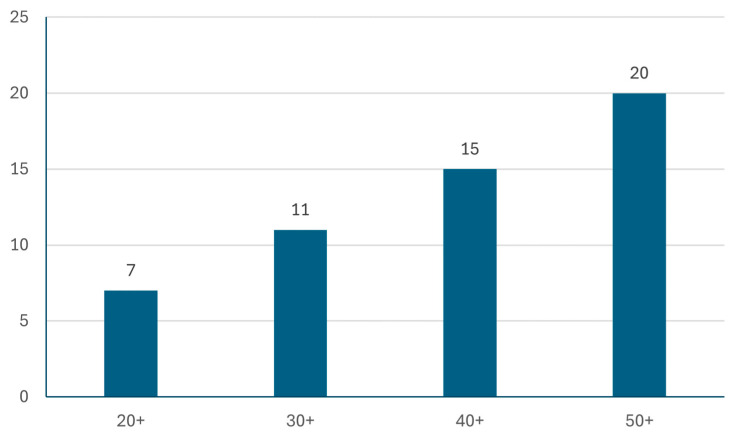
The age distribution of the participants.

A sample of 53 participants was set up for our testing purposes and to find answers to our research questions. The identity of the participants cannot be tracked, but for statistical reasons, we collected their age, gender, and STEM orientation status (Table 2, Figure 5). All the participants are connected to our university and most of them are employees of various faculties in various positions. Some of the participants have weaker connections to the university, such as former students who were working somewhere else at the time of the test.

### 2.4. Evaluation

For the purpose of the present study, it was mainly the video files which were analyzed. Two members of the research group worked together to help and correct each other, make reliable decisions, and set up standards. A spreadsheet was prepared for each participant where the recognizable and separable actions (atomic steps) were recorded along with the time stamp [27]. Based on the number of steps and the time stamps, the entropy of the process was calculated [27,28,29]. The method is a qualitative evaluation, so working in a pair can increase the correctness of the evaluation. Since, according to our knowledge, a similar evaluation system has never been applied prior to our study, setting up a consistent system required several modification phases before a semi-objective IRD (independent, randomly distributed) model was set up.

It was not the central question of the study whether the tasks were completed or not, but the analysis revealed that several participants ignored or were not able to fulfill the requirement that the appearance of the selected paragraph should match the sample figures in the instruction PDF file (Figure 4). This implies that the layout errors of the string of the erroneous document should be eliminated, and then a left indention formatting should be applied to the paragraph. Unlike generally accepted tests, these requirements were not listed (computer cooking) [66], but participants were assumed to be able to reveal and somehow solve them. Consequently, tasks in which the following corrections and formatting were applied are considered to be completed:

- Completing the tasks listed in the instruction PDF file: typing the 13-character long string and changing the font size (Figure 4);
- Deleting the incorrect multiple Space characters (Figure 1 and Figure 2);
- Deleting the incorrect end-of-line paragraph marks (Figure 1 and Figure 2);
- Formatting the paragraph with a left indentation (Figure 3).

Considering the type of activities, the three categories of lean [40,57,58,59,60,61] are used to reveal the nature of the steps.

- Value added (VA);
- Typing the requested 13 + 1 characters;
- Changing the font size;
- Requested but non-value-added (RNVA);
- Positioning the cursor for typing;
- Selecting the paragraph to change the font size with a double click on the selection bar;
- Saving and closing the Word document;
- Non-value-added (NVA);
- Selecting the extra Space characters, positioning the cursor, or setting up a replacement for deleting the extra Space characters;
- Deleting the extra Space characters, changing the alignment, or deleting with replacement;
- Positioning the cursor to delete the extra Space characters;
- Deleting the extra Enter characters;
- Selecting the paragraph to change the font size by dragging the mouse;
- Additional atomic steps carried out by the participants.

We are aware that the selected string (four paragraphs in the erroneous document) contains further syntactic, semantic, typographic, formatting, and stylistic errors (Figure 1 and Figure 2). However, these errors do not affect the application of the font size, the left indent, and the typing, and are far beyond the scope of the present study. In general, documents were recognized as completed when they had neither less nor more formatting and typing/deleting than required.

In addition to the sample of 53 participants, one of the members of our research group was appointed as an etalon. Their document is used to demonstrate a correct and efficient solution to each task. The results of the etalon files are presented in Table 3.

The etalon solution for Tasks 3 and 4 only has VA and RNVA atomic steps, which are in complete accordance with the definition of the properly edited text. For Tasks 1 and 2, NVA steps were required, namely (a)–(d), which do not fulfill the requirements of the properly edited text, and as such generate waste. Participants carried out further NVA atomic steps, which required more information and generated more waste. To prove these statements, the following hypotheses are set up.

### 2.5. Hypotheses

The hypotheses are meant to reveal differences and/or similarities in the processes carried out in the erroneous and proper documents and to calculate and interpret the entropy of the participants’ processes.

1. Modifying erroneous natural language digital texts requires more time and information than their correct correspondents;
2. The increasing number of actions increases the information content required to complete the tasks;
3. The same number of actions with different entropies can reveal the uncertainties of the processes.

### 2.6. Ethical Issues

All participants, who were primarily university employees, teachers, or former students at the university, were sought out and asked to participate in the study individually. They all were also individually informed of the purposes and the process of the test in accordance with the requirements of CUREG [62,67]. It was emphasized that the focus was on problem-solving strategies and not strictly on the results, unlike in widely accepted tests. For the record, most participants were surprised and found it strange that we were primarily interested in the problem-solving processes, unlike tests which they were familiar with.

After discussing the goals and the methods of the testing process, participants worked on the tasks alone, but in the company of one of the members of our research group to provide technical help. The same group member conducted all the testing processes to ensure anonymity and the consistency of the test. The recording application was started and stopped by the participants, allowing them space to quit whenever they felt uncomfortable. Furthermore, after completing the test, participants were allowed to check their recorded files and were allowed to withdraw from the study if they changed their mind.

## 3. Results

In the first step of the analysis, the video file of each participant was broken down into atomic steps [27]. These steps were categorized and recorded in an Excel spreadsheet along with the times at which the step was started and ended. The recording of the atomic steps and the time stamps assigned to them do not include those activities where participants read and checked the instruction PDF file (Figure 4). Strictly, only activities which were carried out in the Word program and interface are taken into consideration. Considering all these requirements, the number of atomic steps, the processing time, and the entropy of the process were calculated for each task and for each participant.

Table 4 and Table 5 show the average, shortest, and longest times and steps, and the entropy in which participants worked on the four tasks. Furthermore, the numbers of completed tasks are shown in Table 4.

In the etalon test, typing the 13-character long string required 8.15 s and 4,72 s in Tasks 1 and 3, respectively (average is 6.435 s). Changing the font size took 3.01 s and 1.44 s (the average is 2.225 s) (Table 3). Similar differences were detected in the participants’ tests (Table 4). We can conclude that typing took more time than changing the font size. This can explain the time difference between Tasks 3 and 4.

However, for the participants, Task 1 required 1.5 times more time than Task 2, the difference (d) being 52.52 s, which is much longer than the time required for typing (Table 4). Further testing and analyses are required, but one of the principles of the lean production system can provide an explanation for this discrepancy [40,59]. Task 1 precedes Task 2 and, as such, can serve as a training task. This finding is in accordance with one of the principles of the lean production system, which states that errors are to be learned from and to be avoided in upcoming tasks and problems [40,57,58,59,60,61]. In our testing environment, this finding implies that the layout errors—shared by Tasks 1 and 2—were discovered in Task 1 and were easier and faster to identify and handle in Task 2.

In the case of Tasks 3 and 4, the time difference between them is much less compared to Tasks 1 and 2, but still greater than the time required for typing. The analysis of the video files revealed that, after completing Task 3, participants were surprised to be ready in such a short time, and spent more time checking the task description, checking it several times (this time is not included but increases the number of steps), or navigating the Word document. In Task 4, they were not that surprised and closed the file in a shorter time than in Task 3.

In the following phase of the analysis, the results were compared regarding the functions of the independent variables: orientation (STEM vs. non-STEM), gender (female vs. male), and age, forming four groups (20+, 30+, 40+, and 50+, 1, 2, 3, and 4, respectively).

**Figure 6:**
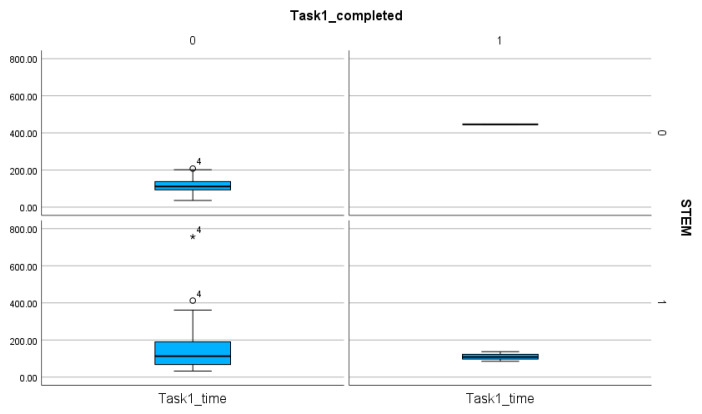
The comparison of the STEM and non-STEM participants on the number of completed and non-completed tasks and the time spent on these solutions in Task 1. The sings (★, ◯) next to the numbers indicate the values outside of the boxplot.

**Figure 7:**
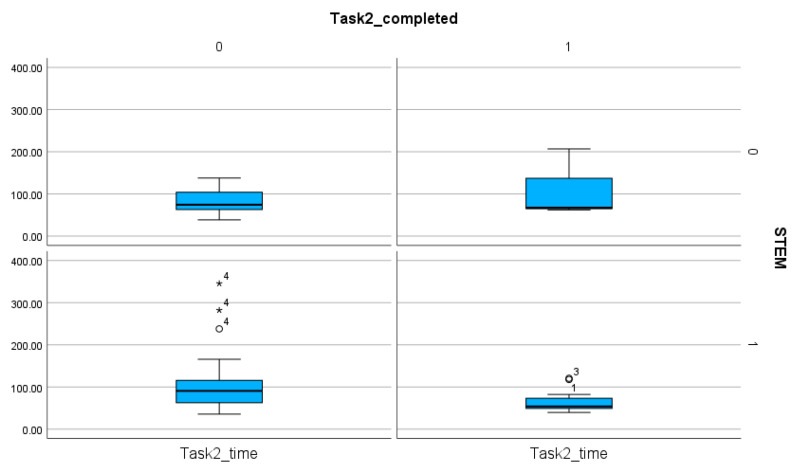
The comparison of the STEM and non-STEM participants on the number of completed and non-completed tasks and the time spent on these solutions in Task 2. The sings (★, ◯) next to the numbers indicate the values outside of the boxplot.

**Figure 8:**
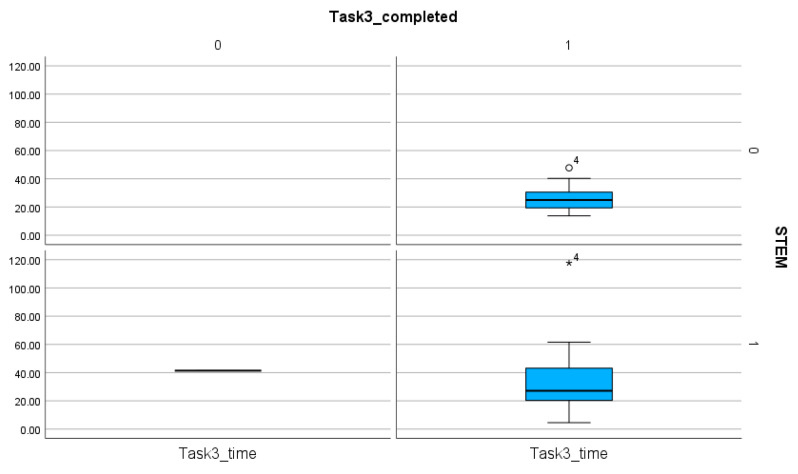
The comparison of the STEM and non-STEM participants on the number of completed and non-completed tasks and the time spent on these solutions in Task 3. The sings (★, ◯) next to the numbers indicate the values outside of the boxplot.

**Figure 9:**
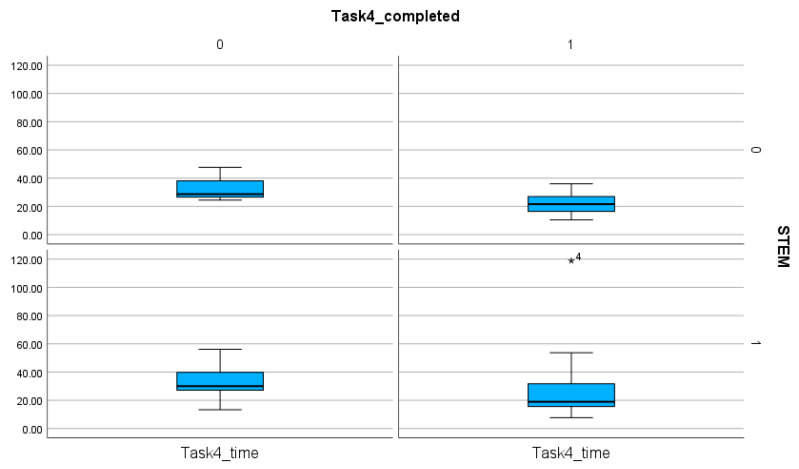
The comparison of the STEM and non-STEM participants on the number of completed and non-completed tasks and the time spent on these solutions in Task 4. The sing (★) next to the number indicates the value outside of the boxplot.

The number of completed tasks is extremely low in Task 1, with only 4 participants able to fulfill all the requirements of the task (Table 4). The comparison of the time spent on the tasks proves that, regardless of the orientation, Tasks 1 and 2 required more time and steps than Tasks 3 and 4. Statistical analyses (T-statistics) show no significant difference between the STEM and the non-STEM subgroups (*p* value for Task 1, Task 2, Task 3, Task 4: 0.412, 0.16, 0.068, 0.347) (Table 6, Figure 6, Figure 7, Figure 8 and Figure 9). This finding proves the presence of efficiency islands [61], the phenomenon where one is a professional in a specialized subject of CS/STEM while underqualified in others and cannot see the connections between the subfields of CS/STEM. One of the consequences of the efficiency island effect is that professionals in informatics in an end-user role cannot apply knowledge gained in studies in informatics and CS/STEM.

In a similar way, there is no significant difference between the STEM and the non-STEM subgroups regarding the number of steps (*p* values for Task 1, Task 2, Task 3, Task 4: 0.367, 0.339, 0.082, 0.246) (Table 7). These findings imply that STEM orientation, including professionals in CS, is not a guarantee of effective digital text management.

In Task 2, 16 completed tasks were found (Table 4). This result can be explained by the training role associated with Task 1. However, the videos revealed that there is another explanation which has a strong connection to the four types of problems [68], which are grouped as follows.

- Reactive;
- Troubleshooting (Type 1);
- Gap from standard (Type 2);
- Proactive;
- Target condition (Type 3);
- Open-ended (Type 4).

**Figure 10:**
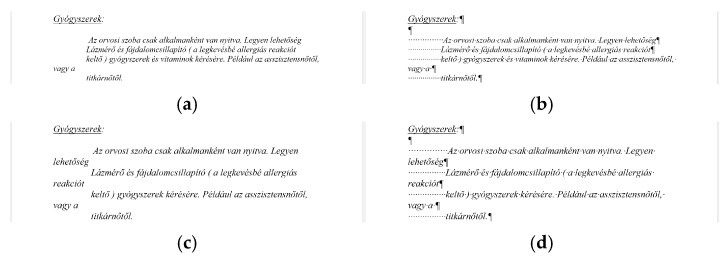
The effect of Type 1 Troubleshooting problem-solving approaches in Tasks 1 and 2. The erroneous text after typing “és vitaminok” in Task 1, the Show/Hide button is turned off/on (**a**)/(**b**). The erroneous text after changing the font size in Task 2, the Show/Hide button is turned off/on (**c**)/(**d**).

All but one of the participants started Task 1 with typing, without cleaning the layout errors from the text. This approach implies that participants preserved the errors until they ran into a breakdown of the text (Figure 10). Furthermore, most participants did not turn on the “Show/Hide” button; consequently, the non-printable characters remained hidden (Figure 10a,c).

Considering the four types of problems [68], this approach belongs to the Troubleshooting (Type 1) category, where only visible errors are handled, without looking for the root causes. Looking for root causes would lead us to Type 2 Gap-from-Standard problem-solving, where the standard is set up by the definition of the properly edited digital text and the collection of rules (standards) regulating these natural language digital texts (error categories are detailed in [3,29]). To reveal these gaps, the andons (visible tools designed to alert operators and managers of problems in real time) [40,59] in the word processor can be a useful data supplier. Most of the andons of the word processor are permanently displayed on the interface—such as menus, tool bars, rulers, dialog panels, and the mouse pointer–indicating their on/off status. However, one of the most important andons, the non-printable characters (Figure 1, Figure 2, Figure 3 and Figure 10b,d), can only be displayed when the end-user finds them useful and necessary. If the Show/Hide button is turned off, the only sign of layout errors in Task 1 is the misplacement of the string “vagy a” after typing the requested “és vitaminok” string (Figure 10a). On the other hand, changing the font size to 16 pt misplaces all the fake paragraphs in Task 2 (Figure 10c), making the errors more obvious. If the root causes of the errors had been revealed in Task 1, the misplacement of the fake paragraphs could have been avoided in Task 2.

We can conclude that both Type 1 and Type 2 problems are reactive. However, to improve the effectiveness of end-user problem-solving and digital artifacts, at least Type 3 Target Condition problem-solving approaches should be employed in real-world situations, like the one presented in this study. The tasks of the test also allow space for Type 3 Target Condition problem-solving, which allowed the participants to not only survive (bricolage) [41,42] but also to present effective solutions. In the sample, there is only one participant who applied Type 3 problem-solving, and altogether, only four participants completed Task 1. The analysis revealed that these four participants were previously trained with the Error Recognition Model described in [69], which further proves the effectiveness of the method.

One further misconception circulating in end-user computing is that non-printable characters are inconvenient or annoying for end-users and should not be presented on the screen [3,4,5,27,28,29,70,71,72,73]. Some participants wanted to delete Space marker dots, while others misinterpreted them as periods. As clearly explained and expressed in the lean production system, andons are there to help reveal errors and handle problems [40,57,58,59,60,61]. In the case of digital text management, non-printable characters can serve as an andon and help end-users to handle layout errors in the text if they are trained how to handle and use them [69]. Their role is magnified in erroneous documents like those in Tasks 1 and 2.

**Figure 11:**
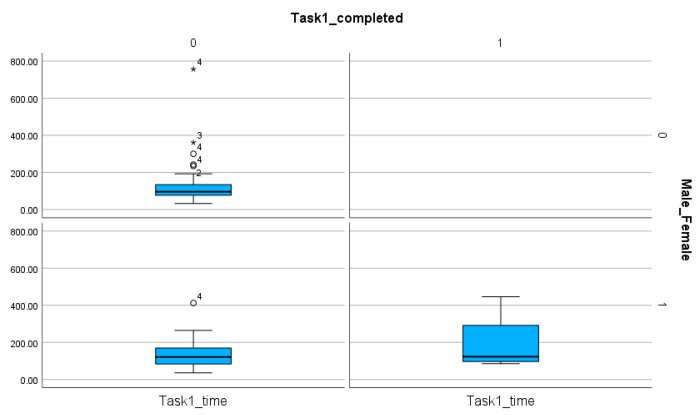
The comparison of male and female participants based on the number of completed and non-completed tasks and the time spent on these solutions in Task 1. The sings (★, ◯) next to the numbers indicate the values outside of the boxplot.

**Figure 12:**
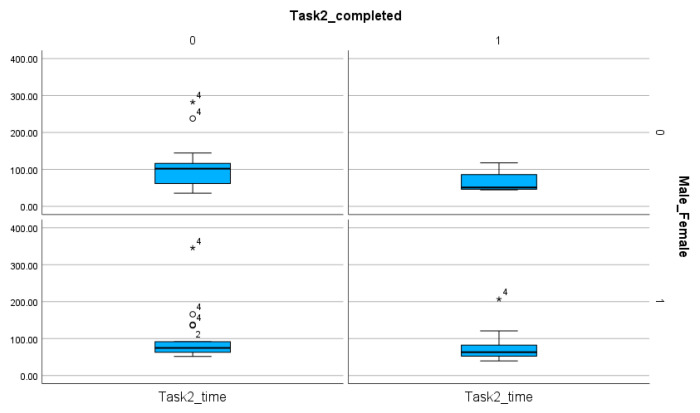
The comparison of male and female participants based on the number of completed and non-completed tasks and the time spent on these solutions in Task 2. The sings (★, ◯) next to the numbers indicate the values outside of the boxplot.

**Figure 13:**
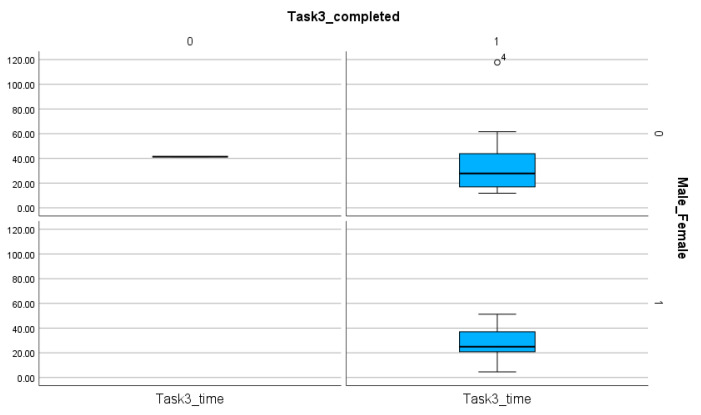
The comparison of male and female participants based on the number of completed and non-completed tasks and the time spent on these solutions in Task 3. The sing (◯) next to the number indicate the value outside of the boxplot.

**Figure 14:**
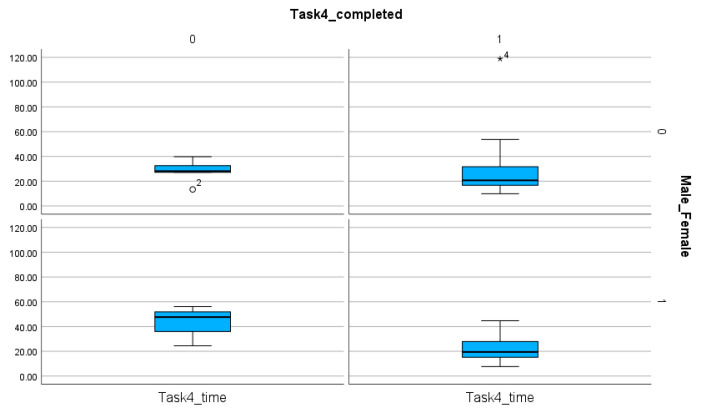
The comparison of male and female participants based on the number of completed and non-completed tasks and the time spent on these solutions in Task 4. The sings (★, ◯) next to the numbers indicate the values outside of the boxplot.

The comparison of female and male participants does not reveal significant differences when the time (*p* value for Task 1, Task 2, Task 3, Task 4: 0.275, 0.231, 0.34, 0.279) (Table 8) and the number of steps (*p* value for Task 1, Task 2, Task 3, Task 4: 0.137, 0.244, 0.46, 0.386) (Table 9) are analyzed. However, the number of completed tasks shows differences (Table 4 and Table 8, Figure 11, Figure 12, Figure 13 and Figure 14). The percentages of completed tasks by females and males are 14.29% (F) and 0% (M) in Task 1, 35.71% (F) and 24% (M) in Task 2, 100% (F) and 96% (M) in Task 3, and 82.86% (F) and 76% (M) in Task 4 (Table 8).

The time spent on the tasks and the number of steps carried out in the completed and uncompleted tasks show a unique pattern. In Task 1, completing the assignment took more time than leaving it unfinished (defect) or overprocessed, which are two of the eight wastes of lean (non-completed in our terms) [59]. However, in Task 2, handling the non-completed documents took more time than the completed ones. The same is true for Tasks 3 and 4. We can conclude that those participants who could complete the tasks were able to learn from their experience in Task 1 and applied this knowledge in the subsequent tasks. Conversely, those participants who could not complete the task do not seem to be aware of their processes, despite their results improving from Task 1 to Task 2 (Table 10 and Table 11).

**Figure 15:**
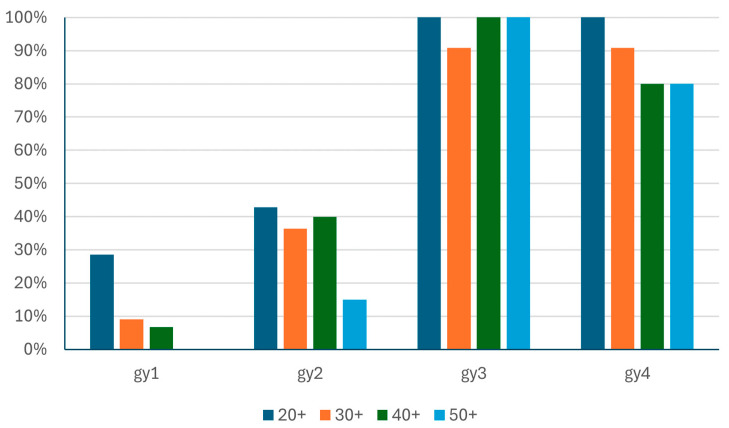
The percentage of completed tasks grouped by age.

**Figure 16:**
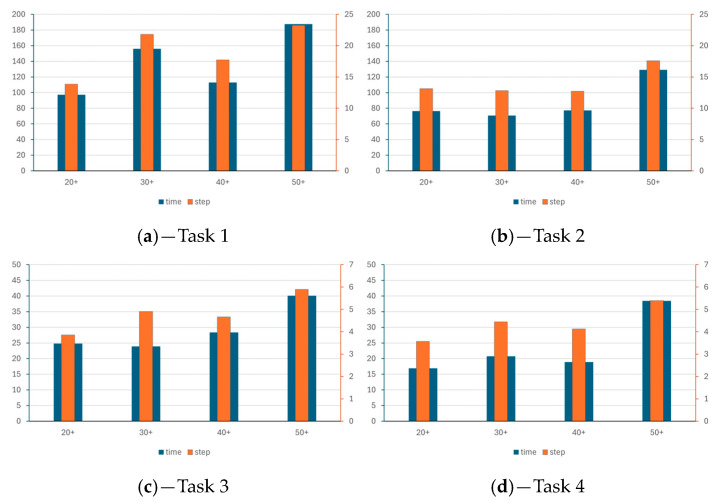
The comparison of time and steps in the different age groups. The left axis indicates the average time spent on the task, and right axis the average number of steps. The subfigures stand for Tasks 1–4, (**a**–**d**) respectively.

Considering the age difference, due to the low number of participants, we cannot draw firm conclusions, but the tendency based on our testing is that young people performed better than older participants (Figure 15 and Figure 16). However, at this point, we must call attention to the fact that the four participants who completed Task 1 had studied with the Error Recognition Model (ERM) described in [69], which is a novel approach that was not available for the older groups. In Task 2, where 16 completed solutions were found, five participants studied with the ERM and 13 were STEM-oriented. It can be assumed that STEM studies might have a positive impact on increasing levels of problem-solving. However, further testing and various groups are needed to confirm age-, STEM-, and ERM-related findings.

We checked by one-way ANOVA for a significant difference in time and the number of steps among the distinct age groups. Table 12 shows the significance values (*p*-values).

We can see in Table 12 that there is only one significant difference between the four age groups in the time taken for Task 4. In this case, we get an eta-square value of 0.151, implying a small effect. In all other cases, there is no significant difference. The Tukey post hoc test results reveal a significant difference between the 40+ and 50+ groups. In all other cases, there is no significant difference. The comparison of the age groups reveals that, in general, the 50+ group needed the longest time to carry out a step (Table 13). However, there are instances where the 20+ group needed more time than the 30+ and 40+ groups. These differences are not significant, but they might be a warning.

The time divided by steps reveals how much time was required to carry out one step on average. A longer time was needed in Task 1 compared to Task 2, and in a similar way, the steps were carried out more slowly in Task 3 than in Task 4. Comparison of the age groups also reveals that the 50+ group needed more time to carry out the steps than the younger groups.

In terms of completing the tasks without any defects or overproduction, the 20+ age group has the best results compared to the others (Table 14). However, the small number of completed tasks in Tasks 1 and 2 and the small number of participants require further testing to find proof for our suggestion.

## 4. Implications and the Limitations of the Study

As mentioned, one of the goals of the study was to set up an evaluation system that can handle natural language digital texts and evaluate end-user activities. It is without question that triangulation [65,67] must be used to cover as many aspects of the problem as possible. However, we can conclude that both the qualitative and the quantitative methods mentioned in the paper can serve as standards [40,57,58,59,60,61], which implies that further research is needed to improve the effectiveness of the method [40,57,58,59,60,61].

One limitation of the process is the evaluation of the video files, which is carried out by humans. We were not able to find any automated solutions to decide on the boundaries—the beginning and end of an atomic step—of unpredictable human actions. AI might solve the problem in the near or far future, but these manual evaluations will be needed to teach AI systems. At present, we use a manual evaluation method to record atomic steps and calculate their time stamps. Later, these recorded data might be used as training data for automated evaluation [74]. Another source of uncertainty is the automated evaluation of the four output files. Building algorithms for unpredictable events and analyzing unknown or non-existent text is beyond the scope of our evaluation system.

One of the most common unpredictable human movements recognized in the video file is moving the mouse aimlessly on the interface. This type of movement was named ‘empty’. The problem with the ‘empty’ atomic step is that it is almost impossible to define its boundaries since there are no distinguishable characteristics to indicate the beginning and the end. In a similar way, when the beginning and/or the end of the string disappeared from the selected paragraph of the Word documents, it is extremely demanding and far beyond the scope of the present study to handle these cases.

Further unexpected actions can be listed to show how unpredictable end-users can include the following:

- Changing the normal style of the whole document;
- Deleting erroneous characters in the whole document, including extra Space characters within parentheses;
- Inserting the green rectangle with rounded corners presented in the instruction PDF to Tasks 1 and 3;
- Applying right indentation to the paragraph;
- Turning off the Window/Orphan control;
- Changing the line spacing, etc.

As mentioned, our research group set up the standard both for the solution of the tasks and the evaluation. However, the recording revealed solutions that we had never encountered before. One of these interesting solutions is the deletion of multiple Space characters by changing the alignment of the paragraph. Another unexpected solution is that the alignment of the paragraph can be changed by selecting the current alignment of the paragraph: if the alignment of the paragraph is left, right, or center, then one clicks on the same alignment button, the alignment of the paragraph is changed to justified, and the other way around; if the alignment is justified and one clicks on the justify button, the alignment of the paragraph is changed to left).

Deleting multiple Space characters by changing the alignment is a fast solution, and we really liked it. In the etalon files, we tried this method and recorded it. This solution shortened the overall time of the modification. However, this solution can only be effective in short texts like the paragraph selected for the test. Considering both the advantages and the disadvantages of deleting multiple Space characters, this solution allows space for redefining the standard, as it is one of the principles of the lean production system and is key to continuous improvement [40,57,58,59,60,61].

One further limitation of the study is the number of participants. Further testing with larger groups should be carried out. However, we found that the following causes create huge obstacles to collecting clear data:

- Willingness to participate in such tests is extremely low;
- The anonymity requirement does not allow us to collect data on orientation, gender, age, and other personal information;
- Several institutes cannot fulfill the technical requirements of the test (e.g., lack of LAN or online classroom in use, insufficient room for saving log files on computers, etc.;
- Low-level computation thinking skills among participants (e.g., participants do not know the difference between Save and Save As, how to compose correct folder and file names, how to compress and extract files, how to download and upload files in online classrooms, how to send large files, etc.).

Furthermore, qualitative analysis of the collected data uses up an enormous amount of human resources, which are limited in our research group. This implies that further research groups should be involved to obtain comparative results.

## 5. Discussion

A group of 53 participants were tested on their problem-solving strategies in erroneous and correct natural language digital texts. With the approval of the participants, three categories of personal data were collected: gender, age, and STEM orientation. However, the identification of the participants cannot be tracked, since the results presented in the paper do not refer to them individually.

To carry out the test, four documents were presented in which one short paragraph needed to be modified according to the task descriptions and the figures accompanying the tasks. The activities of the participants were recorded in two different file formats. In text files, the keyboard and mouse activities were recorded, while in a video file, the entire screen was recorded using a dedicated application called ANLITA (Atomic Natural Language Input Tracker Application). The output of the test consists of six files; two recordings and four Word documents modified by the participants.

To analyze the output files, both quantitative and qualitative methods were applied (triangulation). In the present study, the results of the video analysis are detailed. The analysis was carried out and continuously improved by two members of our research team to set up standards for both the present and further analyses. The solutions of the participants were broken down into atomic steps, which were categorized. Then, the beginning and the ending time of these steps were recorded (time stamp). Based on the time stamps, the probability of the atomic steps, and finally the entropy of the process, were calculated. On the one hand, the calculated entropy reveals the information content required to carry out the process. On the other hand, when the IRD entropy is compared to the IID entropy, we can identify those activities for which information content is high or low.

We have found that if the information content of an atomic step is low compared to the other steps, it indicates uncertainty or allows space for recognizing further steps into which the defined atomic step can be broken down. This latter option can reveal the nature of uncertainty and lack of knowledge, increasing the entropy of the process and the number of NVA activities. In general, it can help to identify the root causes of waste generated in these modification processes.

## 6. Future Research

The present study revealed that compared to an optimized solution (Table 3), where no waste is generated during the problem-solving processes, participants needed far more time, steps, and information than was expected. Our primary concern is to reveal how entropy can vary compared to an IID model and whether these differences allow us to investigate end-users’ uncertainty and inactivity. The goals of the future studies are to find explanations for the losses generated by end-users, call attention to the magnitude of the problem, and to provide guidance for education in the process of finding more effective and efficient approaches and methods for handling natural language digital texts.

For future studies, we plan to use the sample presented and detailed in the current paper, based on the recorded log files, Word documents, and already counted entropies. Furthermore, we plan to find similar, semi-anonymized samples and repeat the investigation in similar conditions to further test the novel EDT method.

Our ultimate goal is to test schools, including both students and teachers, to reveal their problem-solving processes. If the test reveals discrepancies, we can offer immediate interventions before automatic, error-prone processes have a negative impact on the development of the students of the next generation.

## 7. Conclusions

The analysis of 4 × 53 modification processes in erroneous and correct Word documents revealed that the modification of erroneous documents generates waste and requires more human and machine resources than their correct correspondents. It is also proven that erroneous documents can serve as training documents, which implies that end-user education should pay more attention to real-world problem-solving, including erroneous digital artifacts.

In general, we can conclude that fundamental changes are needed in digital education. The widely accepted tool-centered approaches should be left behind to reduce the waste generated by end-users in their data management processes and erroneous digital artifacts. It was also found that problem- and human-centered approaches should be introduced to eliminate waste and find a balance between flow and resource efficiency.

## Figures and Tables

**Table 1:** The number of paragraphs and characters of the selected string in the original erroneous document and in the correct version.

Title 1	#Paragraphs	#Characters with Space	#Characters without Space
original	4	266	179
correct	1	202	178

**Table 2:** The number of participants, their gender, and their STEM orientation status.

	Female	Male	All
STEM-oriented	17	21	38
non-STEM	11	4	15
All	28	25	53

**Table 3:** The results of the etalon in the four tasks and the entropy of the IID (independent, identically distributed) model based on the steps carried out in the problem-solving process.

Task	Time	Steps	Entropy	IID
gy1	26.32	8	2.6187	3.0000
gy2	23.56	9	2.9225	3.1699
gy3	8.03	3	1.3803	1.5850
gy4	4,95	3	1.5426	1.5850

**Table 4:** The average time, the number of steps, and entropy of the four tasks and the number of completed tasks (N = 53).

Task	Time	Steps	Entropy	Completed
gy1	147.83 s	20.04	3.6522	4
gy2	95.31 s	14.55	3.2543	14
gy3	31.39 s	5.08	1.9564	52
gy4	26.39 s	4.60	1.8887	44

**Table 5:** The shortest and longest times and the minimum and maximum number of steps in the four tasks (N = 53).

Task	Time Min	Time Max	Step Min	Step Max
gy1	32.30 s	756.24 s	8	71
gy2	35.75 s	345.40 s	5	71
gy3	4.52 s	117.68 s	3	15
gy4	7.67 s	118.80 s	3	10

**Table 6:** The average time required to finish the four tasks in the STEM- and the non-STEM-oriented groups. The numbers in parentheses indicate the number of completed tasks.

Task	STEM (N = 38)	non-STEM (N = 15)
gy1	150.19 s (3)	140.96 s (1)
gy2	97.69 s (13)	89.30 s (3)
gy3	33.21 s (37)	26.47 s (15)
gy4	27.28 s (32)	24.13 s (13)

**Table 7:** The average number of steps required to finish the four tasks in the STEM- and the non-STEM-oriented groups.

Task	STEM	Non-STEM
gy1	20.50	18.87
gy2	15.13	13.07
gy3	5.16	4.80
gy4	4.58	4.67

**Table 8:** The comparison of female and male participants based on the time spent on Tasks 1–4. The numbers in parentheses indicate the number of completed tasks.

Task	Female (N = 28)	Male (N = 25)
gy1	144.42 (4)	151.11 (0)
gy2	92.37 (10)	98.62 (4)
gy3	28.52 (28)	34.42 (24)
gy4	23.77 (25)	29.33 (19)

**Table 9:** The comparison of female and male participants based on the number of steps carried out in Tasks 1–4.

Task	Female	Male
gy1	18.89	21.32
gy2	14.71	14.36
gy3	4.71	5.44
gy4	4.43	4.80

**Table 10:** The time spent on the completed and non-completed tasks.

Task	Completed	Non-Completed
gy1	194.38 s (4)	143.75 (49)
gy2	76.62 s (14)	102.01 (39)
gy3	31.11 s (52)	41.38 (1)
gy4	25.04 s (44)	33.00 (9)

**Table 11:** The number of steps carried out in the completed and non-completed tasks.

Task	Completed	Non-Completed
gy1	22.25 (4)	19.86 (49)
gy2	10.79 (14)	15.90 (39)
gy3	4.94 (52)	11.00 (1)
gy4	4.55 (44)	4.89 (9)

**Table 12:** One-way ANOVA results between the four age groups. Bold indicates the significant difference.

Tasks & Subject	F (3.48)	Significance (*p*-Value)
Task 1 time	0.625	0.603
Task 1 steps	0.572	0.636
Task 2 time	1.092	0.362
Task 2 steps	1.943	0.135
Task 3 time	1.226	0.310
Task 3 steps	2.398	0.080
Task 4 time	2.836	**0.048**
Task 4 steps	0.821	0.489

**Table 13:** The ratio of the time and the steps of the age groups.

Task	20+	30+	40+	50+
gy1	7.33 s	7.15 s	6.36 s	8.06 s
gy2	6.14 s	5.51 s	6.06 s	7.34 s
gy3	6.49 s	4.87 s	6.07 s	6.80 s
gy4	4.68 s	4.67 s	4.58 s	7.13 s

**Table 14:** The number and percentage of completed tasks in the different age groups.

Task	20+	30+	40+	50+
gy1	2 (29%)	1 (9%)	1 (7%)	0 (0%)
gy2	3 (43%)	3 (27%)	5 (33%)	3 (15%)
gy3	7 (100%)	10 (91%)	15 (100%)	20 (100%)
gy4	7 (100%)	10 (91%)	12 (80%)	15 (75%)

## Data Availability

The original contributions presented in this study are included in the article. Further inquiries can be directed to the corresponding author.
